# Ultra-fast fabrication of MXene/PVA composite films through glutaraldehyde induced microgel framework

**DOI:** 10.1016/j.heliyon.2024.e30714

**Published:** 2024-05-06

**Authors:** Ziwen Gan, Ranran Qi, Bowen Chen, Gaofei Yuan, Mingyi Liao

**Affiliations:** College of Transportation Engineering, Dalian Maritime University, Dalian, Liaoning, China

**Keywords:** Ti_3_C_2_T_x_, Crosslinking, Ti_3_C_2_T_x_/PVA microgel, Self-assembled films

## Abstract

In this study, Ti_3_C_2_T_x_/PVA microgels were assembled through the introduction of glutaraldehyde and PVA into Ti_3_C_2_T_x_ colloids. Subsequently, the microgels underwent vacuum-assisted filtration (VAF) and drying processes to fabricate Ti_3_C_2_T_x_/PVA self-assembled films (MPGF). This research effectively reduced VAF time by introducing a small amount of glutaraldehyde. The findings demonstrate that glutaraldehyde's chemical crosslinking prompts the formation of temporary microgel frameworks between Ti3C2Tx and PVA, enhancing water molecule transfer during VAF and improving film formation efficiency. Further analysis links VAF time is related to the particle size distribution of the microgels. Adjusting crosslinking and PVA quantity alters microgel crystalline structure and –OH hydrogen bonds, affecting particle size and VAF time. Additionally, films produced via rapid VAF exhibit promising mechanical properties for practical applications.

## Highlights

• Glutaraldehyde-induced microgels expedited Ti_3_C_2_T_x_/PVA film fabrication (molding time reduced to 69 s).
• The molding mechanism was explained by investigating hydrogen bonding, cross-linking, and crystal structure transformation.
• MPGF_PVA0.2_ demonstrates a significant 4.7 times improvement in mechanical properties over the Ti_3_C_2_T_x_ film.

## 1 Introduction

Since its introduction in 2011, the two-dimensional (2D) material Ti_3_C_2_T_x_ has garnered significant attention [1,2]. particularly in the context of Ti_3_C_2_T_x_/PVA self-assembled films through Vacuum-Assisted Filtration (VAF) [[3], 4, 5]. Due to their microscopically oriented three-dimensional structure with unique physical and chemical properties, these films hold substantial promise in various applications, including energy storage [[6], [7], [8]], catalysis [[9], [10], [11]], and electromagnetic shielding [[12], [13], [14]]. Nevertheless, the preparation of these self-assembled films encounters challenges related to prolonged processes and high energy consumption, attributed to the 2D "face-to-face" stacking and the mass transfer barrier effect of Ti_3_C_2_T_x_ [15,16].

Two-dimensional materials, exemplified by Ti_3_C_2_T_x_, pose mass transfer challenges in media such as water and air, primarily due to surface van der Waals forces, hydrogen bonding, and geometric effects [[17], [18], [19]]. Wu et al. [20] utilizing density functional theory calculations, simulated the dynamic behavior of water molecules between Ti_3_C_2_T_x_ layers in aqueous environments. The findings suggested that water molecules easily attacked Ti–OH on the Ti_3_C_2_T_x_ layer surface, forming hydrated hydrogen ions, thereby hindering mass transfer. Wen et al. [21] further elucidated this process, demonstrating that the formed hydrated hydrogen ions induced other water molecules between layers to leap stably between adsorption sites, restricting and impeding the transfer and diffusion of water molecules. Muckley et al. [22], in their study, discovered that the presence of other active surface functional groups and ions such as K^+^ and Mg^2+^ in the aqueous phase also promoted surface hydration, affecting mass transfer to varying degrees. Thus, eliminating active functional groups seemed to be an effective strategy for reducing the formation time of Ti_3_C_2_T_x_.

However, as a representative member of the two-dimensional transition metal carbide nitride (MXene) family, Ti_3_C_2_T_x_ is known for its surface rich in active functional groups such as –OH, –F, -Cl, making the elimination of these groups impractical as it would disrupt the unique physicochemical properties of Ti_3_C_2_T_x_. The challenges extend beyond this point. Fan et al. [23], combining VAF and freeze-drying techniques, investigated the mass transfer behavior of water during the formation of Ti_3_C_2_T_x_ films. They observed that due to the high surface area, Ti_3_C_2_T_x_ layers tended to spontaneously aggregate and stack "face-to-face," compelling water molecules to pass only at the edges of the layers, significantly increasing the mass transfer path and difficulty. The VAF formation process typically required several hours. Jin et al. [24] indicated that to prepare composite Ti_3_C_2_T_x_/PVA self-assembled films, the formation time would further increase to several tens of hours because the intercalated PVA occupied more layer space. Iqbal et al. [25] showed that due to the current synthesis methods, Ti_3_C_2_T_x_ layers typically had many defects and vacancies on the surface. Under conditions of water and oxygen, prolonged formation time would inevitably lead to hydration or oxidative degradation. Cao et al. [26] suggested that prolonged exposure to water, air, and light could potentially cause structural transformation of Ti_3_C_2_T_x_ layers, resulting in unnecessary oxidation and performance loss.

Despite these challenges, shortening the formation time is not only advantageous for improving efficiency but also crucial for the stability of material properties in the study of Ti_3_C_2_T_x_, Ti_3_C_2_T_x_/PVA, and other related self-assembled films. Currently, Gao et al. [27] prepared a Ti_3_C_2_T_x_ film with internally foldable structures, which shortened the VAF time. Moreover, these folds can be reconstructed, enabling the film to sensitively capture changes in electromagnetic signals, leading to excellent electromagnetic performance. Wang et al. [28], by using K^+^ to alter the ion balance of the Ti_3_C_2_T_x_ colloidal solution, induced flocculation, and the stacking of flocculated particles during filtration provided suitable gaps for water molecules to pass during solid-liquid separation, avoiding "face-to-face" stacking, thus shortening the film formation time to several tens of minutes. While these works are designed for Ti_3_C_2_T_x_ films, applying them effectively to the formation of Ti_3_C_2_T_x_/PVA self-assembled films is challenging. There is limited research on shortening the formation time of Ti_3_C_2_T_x_/PVA self-assembled films, but the studies have prompted our consideration that avoiding "face-to-face" stacking and providing space and channels for water molecule passage will be the key issue in shortening the formation time of Ti_3_C_2_T_x_/PVA.

For this purpose, our study introduces a highly efficient self-assembly strategy to expedite the production of Ti_3_C_2_T_x_/PVA films (MPGF). The core idea involves leveraging the chemical crosslinking properties of glutaraldehyde to induce the creation of a temporary 3D hydrogel framework between Ti_3_C_2_T_x_ layers and PVA. This framework mitigates the "face-to-face" stacking of Ti_3_C_2_T_x_ layers, establishing pathways and spaces for water flow. Consequently, this approach reduces the VAF formation time, expediting the overall preparation of Ti_3_C_2_T_x_/PVA self-assembled films. Concurrently, an investigation into the optimal dosage of glutaraldehyde and PVA is conducted to determine their impact on the hydrogel framework's specific structure. This analysis includes examining the influence on hydrogen bonding, crosslinking, and crystal structure. The study delves into the mechanism by which the hydrogel influences VAF formation time. Lastly, an exploration of the drying stage's effects on the film's microstructure and mechanical properties is undertaken. Through this comprehensive examination, our research aims to establish both theoretical insights and practical experimental foundations for the application of Ti_3_C_2_T_x_/PVA self-assembled films.

## 2 Experimental

### 2.1 Materials

Titanium aluminum carbide (Ti_3_AlC_2_, 98 %), lithium fluoride (LiF, 99 %), polyvinyl alcohol (PVA, Mw 89000–98000), and glutaraldehyde were purchased from Shanghai Macklin Biochemical Co., Ltd. Hydrochloric acid (HCl, 37 %) was acquired from Tianjin Kemiou Chemical Reagent Co., Ltd. All chemicals were used without further processing.

### 2.2 Design of MPGF

**Fig. 1:**
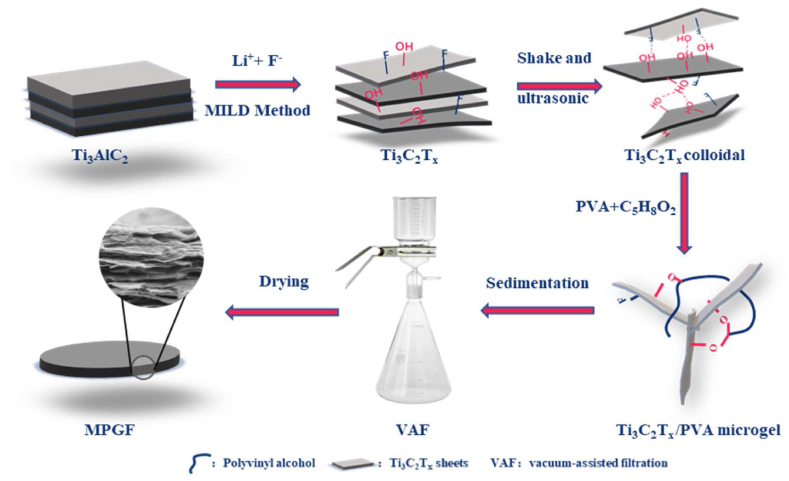
Schematic of the expedited assembly process for MPGF.

The distinctive layered structure and rich surface-active functional groups of Ti_3_C_2_T_x_ sheets facilitate their assembly into films using VAF [29,30]. However, challenges arise from "face-to-face" stacking and blocking effects, hindering mass transfer, prolonging filtration time, and leading to material performance degradation due to oxidation. To overcome these challenges, this study introduces a strategy for preparing MPGF. This is achieved by crosslinking hydroxyl groups on the Ti_3_C_2_T_x_ layer surface with PVA molecules, employing glutaraldehyde to induce the formation of a Ti_3_C_2_T_x_/PVA hydrogel framework, as illustrated in Fig. 1.

Initially, Ti_3_C_2_T_x_ are selectively etched from Ti_3_AlC_2_ phases using LiF/HCl solution. The colloidal solution of Ti_3_C_2_T_x_ sheets is prepared through centrifugation and ultrasonication. Simultaneously, the PVA solution hydrolyzes and fully disperses into the Ti_3_C_2_T_x_ colloid under ultrasonic, resulting in a uniform Ti_3_C_2_T_x_/PVA mixed solution. Subsequently, Glutaraldehyde and hydrochloric acid are added, and gelation takes place under Ar. Following hydrogel formation, the resulting product appears as a flocculent suspension that undergoes layering after settling. Finally, MPGF are obtained through VAF.

#### 2.2.1 Synthesis and delamination of Ti_3_C_2_T_x_

Ti_3_C_2_T_x_ sheets has been prepared by the minimum intensity layer delamination (MILD) approach. Initially, 3.2 g of LiF was dissolved in 40 mL of HCl. Then, 2 g of Ti_3_AlC_2_ powder were slowly added to the previous solution over half an hour, while stirring (350 rpm) at 35 °C for 48 h. After completion of the reaction, the precipitate was washed with 400 mL of DI water and centrifuged at 3500 rpm for 5 min. The washing process was iterated 6 to 8 times to a solution pH of 7. During this iterative process, the supernatant gradually transformed into a dark green colloidal solution upon handshaking as the pH approaches neutrality. Finally, the solution was further delaminated into uniform single-layer and few-layer Ti_3_C_2_T_x_ by sonication for1 h, which was then collected through centrifugation.

#### 2.2.2 Preparation of Ti_3_C_2_T_x_/PVA microgels

In Ar, 10 mL of 0.2 wt% PVA solution was mixed with 100 mL of Ti_3_C_2_T_x_ colloidal solution at 60 °C. 0.02 ml glutaraldehyde and 2 drops of HCl were added to the mixed solution, with stirring for 2 h. Finally, the Ti_3_C_2_T_x_/PVA microgels were prepared. Ti_3_C_2_T_x_/PVA hydrogels with different degrees of cross-linking were obtained by adjusting the amount of glutaraldehyde (0.02 ml, 0.04 ml, 0.06 ml, 0.08 ml). They were labeled as MPGF_GA0.02_, MPGF_GA0.04_, MPGF_GA0.06_和MPGF_GA0.08_. Similarly, Ti_3_C_2_T_x_/PVA hydrogels with varying PVA content were obtained by adjusting the mass fraction of PVA solution (0.1 wt%, 0.2 wt%, 0.3 wt%, 0.4 wt%). They were denoted as MPGF_PVA0.1_, MPGF_PVA0.2_, MPGF_PVA0.3_ and MPGF_PVA0.4_.

#### 2.2.3 Preparation of MPGF

A 40 ml colloidal solution of Ti_3_C_2_T_x_ or Ti_3_C_2_T_x_/PVA hydrogel was film-cast using a vacuum-assisted filtration (VAF) method. The filtration membrane employed was a cellulose blend membrane with a pore size of 0.45 μm and a diameter of 50 mm. Following filtration, the resultant films, specifically the Ti_3_C_2_T_x_ film or MPGF, underwent further drying. Both film types could be easily peeled off from the filtration membrane. It is noteworthy that during the filtration of the Ti_3_C_2_T_x_ colloidal solution, the process was prolonged. And a small amount of white Ti_3_C_2_T_x_ oxide sheets could be observed floating. Conversely, when filtering the Ti_3_C_2_T_x_/PVA hydrogel solution, a rapid stacking of flocculent gel particles. And a swift flow of water through the bottom of the filtration membrane could be distinctly observed.

### 2.3 Characterization

The microstructure and dimensions of Ti_3_C_2_T_x_, Ti_3_C_2_T_x_/PVA hydrogel, and MPGF were investigated using the scanning electron microscope (SEM, Supera-55-sapphire, German). Specimens, precisely sectioned into 0.5 cm^2^ thin slices, were affixed to a conductive adhesive for testing at an acceleration voltage ranging from 10 to 20 kV.

The microstructure of Ti_3_C_2_T_x_ layers and Ti_3_C_2_T_x_/PVA hydrogel powder was observed through the Transmission Electron Microscope (TEM, JEM-F200, Japan). The samples were dissolved in deionized water and subjected to ultrasonic dispersion for 5 min. Subsequently, the dispersed solution was deposited onto a copper grid and left to dry.

The phase and crystal structure of Ti_3_C_2_T_x_ and Ti_3_C_2_T_x_/PVA hydrogel powder were analyzed using the X-ray diffractometer (XRD, D/MAX-Ultima, Japan). Samples, positioned in a 220.1 sample holder, underwent testing with Cu as the X-ray source, within a 2θ range of 3°–50° and a scanning speed of 4°/min.

Surface elemental composition and chemical structure analysis of the samples were performed using the X-ray photoelectron spectroscopy (XPS, ESCALAB Xi^+^, USA). The light source, set at 1486.6 eV with an emission power of 250 W, employed Al Kα X-rays. Casa XPS software facilitated fitting and processing elemental spectra, all calibrated to the C1s binding energy (284.4 eV).

Fourier-transform infrared spectroscopy (FT-IR, Spectrum 3, USA) characterized the bulk chemical structure information of Ti_3_C_2_T_x_ and Ti_3_C_2_T_x_/PVA hydrogel powder. To ensure comparability, 2 mg of the sample was ground together with 50 mg of KBr, resulting in a uniform and transparent pellet compressed at 10 MPa for 5 min. The testing scan range was 4000∼450 cm⁻^1^, with 32 scans and a resolution of 2 cm⁻^1^.

Particle size distribution tests for Ti_3_C_2_T_x_ and Ti_3_C_2_T_x_/PVA hydrogel were conducted using laser particle size analyzer (LPSA, BT-9300SE, Chain). Prepared at a concentration of 1.5 mg/ml, samples were subjected to a sample chamber speed of 4000 rpm, with deionized water added during ultrasonication. The instrument was set for a scanning duration of 180 s, 2 scans and a resolution of 2 s.

For mechanical property assessment, MPGF were analyzed using Universal Tensile Testing Machine (CM, DSA502A, Chain). Following the standards of GB/T 6672-2001 for thin film mechanical measurements, the testing temperature was 25 °C, and the stretching rate was 20 mm/min.

## 3 Results and discussion

### 3.1 Microstructures of Ti_3_C_2_T_x_/PVA microgels

**Fig. 2:**
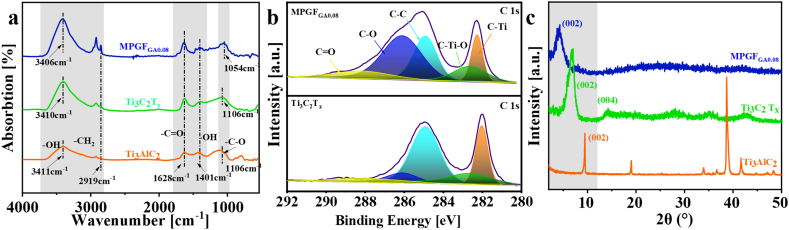
(a) FT-IR spectra of Ti_3_AlC_2_, Ti_3_C_2_T_x_, and MPGF_GA0.8_; (b) XPS C1s fitting spectra of Ti_3_C_2_T_x_, and MPGF_GA0.8_; (c) XRD spectra of Ti_3_AlC_2_, Ti_3_C_2_T_x_, and MPGF_GA0.8_.

To validate the successful preparation of Ti_3_C_2_T_x_/PVA microgels, we initially analyzed the FT-IR spectra of Ti_3_AlC_2_, Ti_3_C_2_T_x_, and MPGF_GA0.8_ microgels were analyzed, as shown in Fig. 2a. Both Ti_3_AlC_2_ and Ti_3_C_2_T_x_ exhibit absorption at 3410 cm^−1^, 2919 cm^−1^, 1628 cm^−1^, 1106 cm^−1^ and 1401 cm^−1^, corresponding to the stretching and bending vibration of –OH, C–H, –C=O, and -C-O bonds, respectively. However, compared to Ti_3_AlC_2_, the –OH peak of Ti_3_C_2_T_x_ significantly increase, while the C–H and –C=O peaks show slight enhancements, indicating the presence of a substantial quantity of –OH and a minimal amount of –C=O after etching. Additionally, there is an alteration in the symmetry of C–H stretching vibration.

In contrast to Ti_3_C_2_T_x_, the intensity of the –C=O bond in MPGF_GA0.8_ microgels increases, and the -C-O bond becomes narrower, shifting from 1106 cm^−1^ to 1054 cm^−1^. This is attributed to chemical cross-linking, resulting in the formation of –C=O and -C-O. Simultaneously, the –OH peak becomes significantly broader and stronger, shifting from 3410 cm^−1^ to 3406 cm^−1^. The –OH peak at 1401 cm^−1^ is stronger and narrower. indicating the formation of numerous hydrogen bonds between crosslinked Ti_3_C_2_T_x_ and PVA, indicating the formation of numerous hydrogen bonds between crosslinked Ti_3_C_2_T_x_ and PVA. the formation of hydrogen bonds between crosslinked Ti_3_C_2_T_x_ and PVA. Furthermore, the C–H peak is also stronger and narrower, suggesting a distinct structural difference between MPGF_GA0.8_ microgels and Ti_3_C_2_T_x_.

XPS analysis was conducted on the surface chemical structures of Ti_3_C_2_T_x_ and MPGF_GA0.8_, as depicted in Fig. 2b. In the C 1s spectra, the appearance of five peaks at 282.0 eV, 282.6 eV, 284.9 eV, 286.1 eV, and 288.8 eV corresponds to C–Ti bonds, C–Ti–O bonds, C–C bonds, C–O bonds, and C=O bonds, respectively [31]. Compared to Ti_3_C_2_T_x_, the surface chemical structure of MPGF_GA0.8_ hydrogels have undergone modifications. The binding energy of C–Ti bonds increased from 282.0 eV to 282.2 eV, the peak value of C–Ti–O bonds at 282.6 eV slightly increased, and the peak value of -C-O bonds at 286.1 eV significantly increased. This suggests the formation of new -C-O bonds and C–Ti–O bonds on the surface of Ti_3_C_2_T_x_, a consequence of the chemical crosslinking between Ti_3_C_2_T_x_ and PVA.

The phase and crystal structures of Ti_3_AlC_2_, Ti_3_C_2_T_x_, and MPGF_GA0.8_ microgels were investigated using XRD, as illustrated in Fig. 2c. Ti_3_C_2_T_x_ exhibits the absence of the Al atomic characteristic peak at 38.68°. The (002) peak also shifts from 9.46° to 4.52°, leading to an increase in interplanar spacing from 0.93 nm to 1.95 nm compared to Ti_3_AlC_2_. This signifies the successful etching of the Al atomic layer, resulting in a layer spacing of 1.95 nm. Conversely, the (002) peak of MPGF_GA0.8_ hydrogels shift further to 4.04° compared to Ti_3_C_2_T_x_, accompanied by an increased interplanar spacing of 2.19 nm. This indicates the successful intercalation of PVA into Ti_3_C_2_T_x_.

**Fig. 3:**
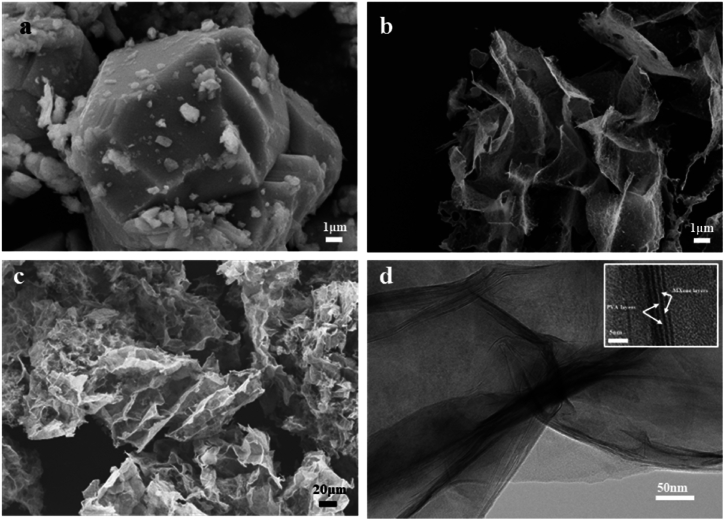
(a) SEM images of Ti_3_AlC_2_; (b) SEM images of Ti_3_C_2_T_x_ sheets; (c) SEM images of MPGF_GA0.8_; (d) TEM image of MPGF_GA0.8_.

The morphological changes of Ti_3_AlC_2_, Ti_3_C_2_T_x_, and MPGF_GA0.8_ was observed by SEM, as shown in Fig. 3. In Fig. 3a, Ti_3_AlC_2_ exhibits a blocky structure. In Fig. 3b, post-etching and delamination, Ti_3_C_2_T_x_ displays a layered structure. In Fig. 3c, MPGF_GA0.8_ microgels present a wrinkled clustered morphology, with a size significantly larger than Ti_3_C_2_T_x_ layers. To further characterize the morphological features of the microgels, TEM was used to observe MPGF_GA0.8_ microgels, as illustrated in Fig. 3d. The Ti_3_C_2_T_x_, with a larger mass thickness contrast, forms a specific framework structure, coalescing with the PVA to assemble a gel-like structure. Local magnification reveals the intercalated structure of PVA, consistent with XRD analysis.

In summary, through glutaraldehyde crosslinking, Ti_3_C_2_T_x_ and PVA are induced to form a 3D hydrogel framework, with PVA layers inserted between Ti_3_C_2_T_x_ layers. This structure differs from the purely layered structure reported in other Ti_3_C_2_T_x_/PVA composite materials [15].

### 3.2 VAF shaping of Ti_3_C_2_T_x_/PVA microgels

To evaluate the efficiency of reducing VAF molding time through the forming of Ti_3_C_2_T_x_/PVA microgels, we initially examined the particle size distribution and corresponding VAF molding time for Ti_3_C_2_T_x_/PVA microgels with different glutaraldehyde amounts. In the study, all comparisons of structure and performance of MPGF are referenced against the original MXene film, as glutaraldehyde and PVA jointly influence the structure and performance of the composite film.

**Fig. 4:**
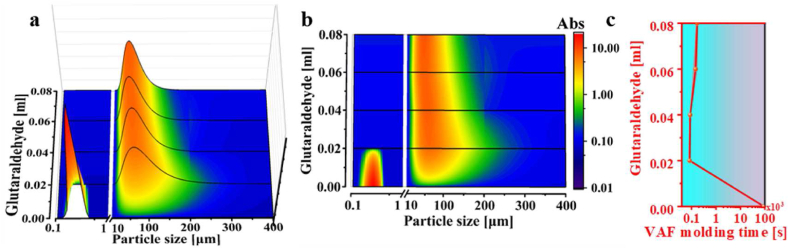
(a) Particle size distribution, (b) mapping of particle size distribution, and (c) VAF forming time curve of Ti_3_C_2_T_x_ and Ti_3_C_2_T_x_/PVA microgels with different glutaraldehyde amounts.

In Fig. 4a and b, the vertical axis represents the Ti_3_C_2_T_x_ sheet colloid solution, MPGF_GA0.2_, MPGF_GA0.4_, MPGF_GA0.6_, and MPGF_GA0.8_ microgels, respectively, with different glutaraldehyde amounts (0 ml, 0.02 ml, 0.04 ml, 0.06 ml, and 0.08 ml).

Compared to the median particle size of 0.28 μm for Ti_3_C_2_T_x_, the particle size of MPGF_GA0.2_ microgels increased by two orders of magnitude, reaching 66.17 μm, accompanied by a broadened particle size distribution. As the glutaraldehyde amount increased, the median particle size decreased to 51.15 μm, and the particle size distribution became narrower.

In Fig. 4c, the average preparation time of Ti_3_C_2_T_x_ membranes is 90400 s, with more detailed data provided in Table S1. In contrast, MPGF_GA0.2_ microgels dramatically reduced this time to 84 s, gradually increasing to 141 s with higher glutaraldehyde amounts. This observation underscores a clear correlation between the glutaraldehyde amount and particle size distribution. Consequently, larger particle sizes and wider distributions in the microgel lead to shortened VAF molding times, and vice versa.

The changes in particle size may be attributed to the chemical crosslinking effect of glutaraldehyde, which can be determined by FT-IR analysis [32]. As shown in Fig. 2a, after the introduction of glutaraldehyde, the –C=O vibration peak at 1401 cm-1 for MPGF_GA0.08_ is enhanced, and the -C-O vibration peak at 1106 cm-1 shifts to 1054 cm-1, indicating the occurrence of chemical crosslinking.

Crucially, after forming the Ti_3_C_2_T_x_/PVA microgel framework, the VAF molding time was reduced by two orders of magnitude. This substantial reduction is attributed to the loose microgel framework, which, during the VAF process, tends to form microscopic route during orientation and stacking. These routes prevent the "face-to-face" stacking of Ti_3_C_2_T_x_, providing space for the rapid passage of water.

**Fig. 5:**
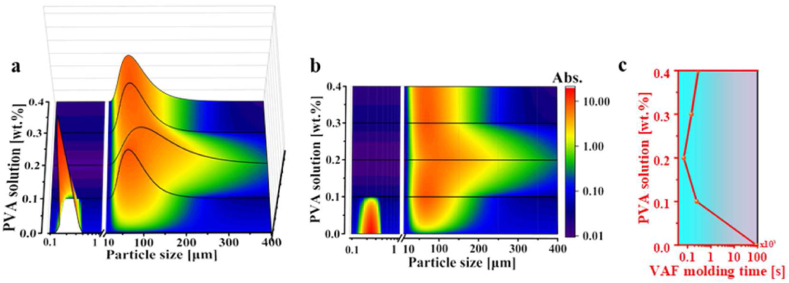
(a) Particle size distribution, (b) mapping of particle size distribution, and (c) VAF forming time curve of Ti_3_C_2_T_x_ and Ti_3_C_2_T_x_/PVA microgels at different PVA concentrations.

Concurrently, we explored the influence of PVA concentration on the particle size distribution and VAF molding time of Ti_3_C_2_T_x_/PVA microgels. As illustrated in Fig. 5a and b, the vertical axis represents the PVA concentration, ranging from 0 wt%, 0.1 wt%, 0.2 wt%, 0.3 wt%, to 0.4 wt%, corresponding to Ti_3_C_2_T_x_ sheet colloid solution, MPGF_PVA0.1_, MPGF_PVA0.2_, MPGF_PVA0.3_, and MPGF_PVA0.4_ microgels, respectively.

In comparison to Ti_3_C_2_T_x_, the particle size of MPGF_PVA0.1_ increased by two orders of magnitude, reaching 64.22 μm, with a widened particle size distribution. As the PVA concentration increased, the median particle size initially rose to 94.19 μm and then decreased to 64.17 μm, accompanied by an initial widening and subsequent narrowing of the particle size distribution.

In Fig. 5c, the film formation time for MPGF_PVA0.1_ microgels was 249 s, whereas MPGF_PVA0.2_ microgels reduced this time to 69 s. Further increase in PVA concentration extended the time to 291 s. Therefore, the VAF molding times also demonstrated a clear correlation with particle size distribution, where larger particle sizes and wider distributions resulted in shortened VAF molding times.

Therefore, the formation of the microgel framework inherently shortens the VAF molding time. By adjusting the dosages of glutaraldehyde and PVA to control the particle size of the microgels, the VAF molding time can be further influenced.

### 3.3 Assembly mechanism of Ti_3_C_2_T_x_/PVA microgels

Given the pronounced influence of Ti_3_C_2_T_x_/PVA microgel particle size on VAF forming time, this is likely associated with factors such as hydrogen bonds, crosslinking, and the crystal structure of the microgels. Consequently, it is imperative to delve deeper into the specific structure and assembly mechanism of Ti_3_C_2_T_x_/PVA microgels.

#### 3.3.1 Dosage of glutaraldehyde

Utilizing FT-IR, XPS, and XRD analyses, we explored the impact of glutaraldehyde dosage on hydrogen bonds, crosslinking, and crystal structure within Ti_3_C_2_T_x_/PVA microgels. The FT-IR spectra of the microgels were scrutinized in the wavenumber range of 3700 cm^−1^ to 3000 cm^−1^, corresponding to the stretching vibrations of –OH.

**Fig. 6:**
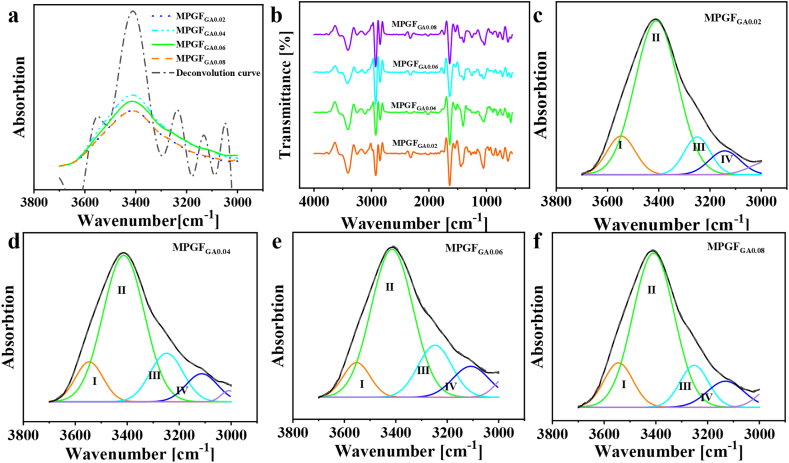
(a) FT-IR and deconvoluted spectra, (b) FT-IR second derivative spectra, and (c–f) fitted spectra of Ti_3_C_2_T_x_/PVA microgels within 3700 cm⁻^1^–3000 cm⁻^1^.

In Fig. 6a, the deconvoluted spectrum of the –OH peak, derived through inverse Fourier transform and the convolution theorem, reveals maxima at 3545 cm^−1^, 3408 cm^−1^, 3250 cm^−1^, and 3120 cm^−1^. In Fig. 6b, the second derivative spectrum exhibits minimum at the same wavenumbers. These findings indicate that the –OH peak in the microgels comprises free –OH (Ⅰ), self-associated –OH (Ⅱ), cyclic –OH (Ⅲ), and –OH…O (Ⅳ) [32,33].这These subpeaks correspond to various types of hydrogen bonds, where self-associated –OH and cyclic –OH represent intramolecular hydrogen bonds within Ti_3_C_2_T_x_ or PVA molecules, and –OH…O hydrogen bonds represent intermolecular hydrogen bonds between Ti_3_C_2_T_x_ and PVA.

The fitted spectra, illustrated in Fig. 6c–f, operate under the assumption that the Fermi resonance effect induced by the –C=O double-frequency vibration at 3260 cm^−1^ to 3250 cm^−1^ is negligible, and that –OH hydrogen bonds are mutually independent. The fitted peak area percentages exhibit a linear correlation with the proportions of hydrogen bonds, with the –OH at the edge of the Ti_3_C_2_T_x_ layer considered negligible. The calculated results, summarized in Table 1, reveal standard deviations within 5.00 %, affirming the reliability of the fitting outcomes.

**Table 1:** Fitting results of –OH hydrogen bonding in Ti_3_C_2_T_x_/PVA hydrogels with varying glutaraldehyde dosage.

Ti_3_C_2_T_x_/PVA type	Hydrogen bond type		Wavenumber [cm^−1^]	Peak area	Relative content [%]	Standard deviation
MPGF_GA0.02_	Free OH	Ⅰ	3546.95	2.01	11.61	0.01
	Self-associated OH	Ⅱ	3409.47	12.09	70.10	0.02
	OH⋯O	Ⅲ	3249.52	1.81	10.49	0.01
	Cyclic OH	Ⅳ	3142.60	1.36	7.81	0.01
MPGF_GA0.04_	Free OH	Ⅰ	3546.10	2.66	11.41	0.02
	Self-associated OH	Ⅱ	3413.54	14.66	63.07	0.04
	OH⋯O	Ⅲ	3248.00	3.86	16.60	0.04
	Cyclic OH	Ⅳ	3113.07	2.14	8.91	0.03
MPGF_GA0.06_	Free OH	Ⅰ	3552.31	2.06	9.37	0.02
	Self-associated OH	Ⅱ	3414.44	13.70	62.30	0.03
	OH⋯O	Ⅲ	3247.68	3.91	17.81	0.03
	Cyclic OH	Ⅳ	3108.52	2.46	10.53	0.03
MPGF_GA0.08_	Free OH	Ⅰ	3544.39	2.32	13.16	0.03
	Self-associated OH	Ⅱ	3408.82	11.46	65.19	0.04
	OH⋯O	Ⅲ	3251.71	2.15	12.21	0.03
	Cyclic OH	Ⅳ	3130.44	1.71	9.44	0.02

As the glutaraldehyde dosage increases, the proportion of self-associated –OH hydrogen bonds decline from 70.10 % to 62.30 %, subsequently rising to 65.19 %. This trend exhibits an initial decrease followed by an increase. Conversely, the –OH…O hydrogen bond content initially ascends from 10.49 % to 17.81 %, then descends to 12.21 %. Cyclic –OH hydrogen bonds follow a similar pattern, ascending from 7.81 % to 10.53 % and then descending to 9.44 %. All demonstrate an initial increase followed by a decrease. The inflection point occurs at MPGF_GA0.06_. This implies that a moderate increase in cross-linking transforms disordered self-condensation –OH hydrogen bonds into locally ordered cyclic –OH hydrogen bonds and intermolecular hydrogen bonds (OH⋯O). This induces local orderliness in the microgel structure and enhancing intermolecular interactions. However, excessive crosslinking disrupts this order.

As shown in Fig. 4a and b, the particle size distribution of Ti_3_C_2_T_x_/PVA changed after crosslinking with glutaraldehyde. Compared to the Ti_3_C_2_T_x_ colloidal solution, the particle size of the microgels formed after the addition of glutaraldehyde and PVA increased by two orders of magnitude, and the particle size distribution became wider. However, with an increase in the amount of glutaraldehyde, the particle size slightly decreased, and the distribution narrowed. This indicates that an increase in crosslinking degree, intermolecular hydrogen bonding, and enhancement of local orderliness result in a reduction in microgel particle size.

Furthermore, the content of free –OH decreases from 11.41 % to 9.37 %, followed by a rebound to 13.16 %. This also indicates in the variation of orderliness. In an ordered structure, the content of free –OH decreases, whereas in the case of excessive cross-linking, free –OH is released again.

The changing pattern of hydrogen bond structures elucidates the changes in Ti_3_C_2_T_x_/PVA microgel particle size. Specifically, a moderate increase in cross-linking enhances the local orderliness of hydrogen bond structures, resulting in volume contraction of microgels in the aqueous environment and consequently a decrease in particle size distribution. Notably, excessive cross-linking disrupts the ordered structure of hydrogen bonds. However, LPSA results indicate that the microgel particle size still decreases, and the distribution continues to narrow. This suggests that the impact of the chemical cross-linking structure on microgel particle size variation is more significant.

**Fig. 7:**
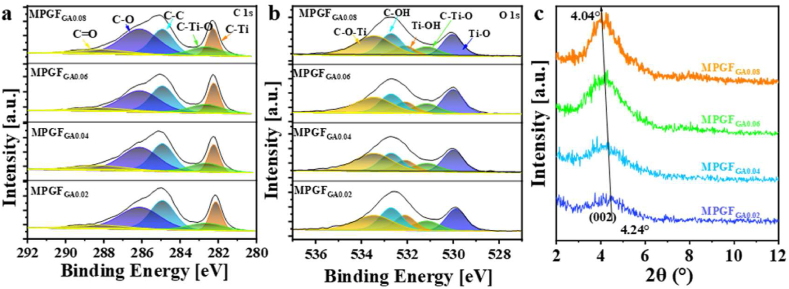
(a) XPS C1s spectra, (b) XPS O1s spectra, and (c) XRD spectra of MPGF_GA0.02_ to MPGF_GA0.08_ microgels.

XPS was employed to investigate the surface structure of Ti_3_C_2_T_x_/PVA microgels. In Fig. 7a, the C1s spectrum reveals that, with an increase in glutaraldehyde dosage, the peaks corresponding to C–O and C–Ti–O at 286.1 eV and 282.8 eV, respectively, gradually intensify. This signifies an augmentation in the quantity of surface C–O and C–Ti–O bonds on Ti_3_C_2_T_x_. Additionally, the binding energy of C–Ti bonds strengthens to 282.2 eV, indicating an outward transfer of valence electrons on the Ti_3_C_2_T_x_ surface. This suggests that the crosslinking degree between the Ti_3_C_2_T_x_ surface and PVA gradually increases with an elevated glutaraldehyde dosage.

In Fig. 7b, an increased glutaraldehyde dosage is observed to enhance the O1s spectrum of the microgels. The peak at 533.4 eV corresponding to C–*O*–Ti intensifies. The binding energy of Ti–O bonds on the Ti_3_C_2_T_x_ surface increases from 529.9 eV to 531.1 eV, while the peak value at 532.0 eV for Ti–OH gradually decreases. This indicates an increased crosslinking degree on the Ti_3_C_2_T_x_ surface, resulting from the growing reaction between Ti–OH on the Ti_3_C_2_T_x_ surface and glutaraldehyde. This explains the disruption of the local orderliness of the hydrogen bond structure in MPGF_GA0.08_ microgels; however, an increase in particle size is not observed due to the limiting effect of excessive crosslinking.

In Fig. 7c, XRD was further employed to investigate the influence of glutaraldehyde dosage on the crystal structure of the microgels. The intensity of the (002) peak increases with an elevation in glutaraldehyde dosage, indicating an enhancement in the orderliness of Ti_3_C_2_T_x_/PVA microgels. Simultaneously, the peak position shifts from 4.24° to 4.04°, and the interplanar spacing increases from 2.08 nm to 2.19 nm. This demonstrates that crosslinking does not hinder further intercalation of PVA.

Through glutaraldehyde crosslinking, a chemical crosslinking network is established between Ti_3_C_2_T_x_ and PVA, inducing changes in hydrogen bonding and crystal structure, PVA further intercalates with Ti_3_C_2_T_x_, ultimately forming microgels with a certain framework structure. Proper addition of glutaraldehyde can enhance the crosslinking degree, hydrogen bonding, and ordering of crystal structure in the Ti_3_C_2_T_x_/PVA microgel framework.

In summary, an appropriate dosage of glutaraldehyde can enhance the crosslinking degree, orderly hydrogen bonds, and crystal structure of Ti_3_C_2_T_x_/PVA microgels. In practical applications, the dosage of glutaraldehyde should be considered. TG analysis (Fig. S1) indicates when used in small amounts, the consumption of active functional groups and PVA components in the film is minimal, and does not significantly affect the activity, composition, and structure of the film, whereas excessive usage may have the opposite effect.

#### 3.3.2 Concentration of PVA

**Fig. 8:**
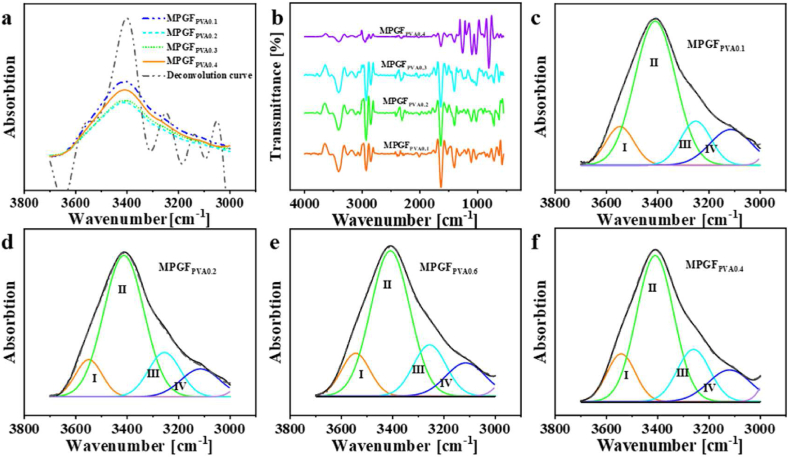
(a) FT-IR and deconvoluted spectra (b) FT-IR second derivative spectra, and (c–f) fitting spectra for MPGF_PVA0.1_ to MPGF_PVA0.4_ microgels within 3700 cm^−1^ to 3000 cm^−1^.

FT-IR, XPS, and XRD analyses were conducted to investigate the impact of PVA content on the hydrogen bond, cross-linking, and crystal structure of Ti_3_C_2_T_x_/PVA microgels. Initially, the –OH stretching vibration peaks in the range of 3700 cm^−1^ to 3000 cm^−1^ were isolated from the FT-IR spectra of MPGF_PVA0.1_ to MPGF_PVA0.4_ microgels. The deconvoluted spectrum, as shown in Fig. 8a, was characterized by maxima at 3547 cm^−1^, 3409 cm^−1^, 3251 cm^−1^, and 3115 cm^−1^.

In Fig. 8b, the second derivative spectrum revealed corresponding minimum at the same wavenumbers, representing free –OH (Ⅰ), self-associated –OH (Ⅱ), cyclic –OH (Ⅲ), and –OH…O (Ⅳ). The fitting results, as illustrated in Fig. 8c–f, were summarized in Table 2, presenting the calculated hydrogen bond content.

**Table 2:** Fitting results of –OH hydrogen bonds in Ti_3_C_2_T_x_/PVA microgels with different PVA contents.

Ti_3_C_2_T_x_/PVA type	Hydrogen bond type		Wavenumber [cm^−1^]	Peak area	Relative content [%]	Standard deviation
MPGF_PVA0.1_	Free OH	Ⅰ	3546.81	1.99	11.21	0.04
	Self-associated OH	Ⅱ	3409.40	10.81	60.88	0.05
	OH⋯O	Ⅲ	3250.82	2.43	13.70	0.03
	Cyclic OH	Ⅳ	3114.68	2.52	14.22	0.05
MPGF_PVA0.2_	Free OH	Ⅰ	3549.19	1.28	10.76	0.11
	Self-associated OH	Ⅱ	3412.46	7.42	62.56	0.12
	OH⋯O	Ⅲ	3254.71	1.85	15.55	0.04
	Cyclic OH	Ⅳ	3115.52	1.32	11.13	0.04
MPGF_PVA0.3_	Free OH	Ⅰ	3544.09	1.59	12.39	0.05
	Self-associated OH	Ⅱ	3409.00	7.45	58.10	0.05
	OH⋯O	Ⅲ	3255.30	2.18	17.03	0.03
	Cyclic OH	Ⅳ	3115.36	1.60	12.48	0.04
MPGF_PVA0.4_	Free OH	Ⅰ	3542.29	1.37	13.78	0.03
	Self-associated OH	Ⅱ	3409.70	10.07	55.17	0.03
	OH⋯O	Ⅲ	3260.18	1.66	17.37	0.06
	Cyclic OH	Ⅳ	312036	1.98	13.68	0.04

With an increase in PVA concentration, the content of self-associated –OH hydrogen bonds initially rise from 60.88 % to 62.56 % and then decreases to 55.17 %, displaying a pattern of initial increase followed by a decrease. Conversely, cyclic –OH hydrogen bonds decrease from 14.22 % to 11.13 %, then increase to 13.68 %. Free –OH decreases from 11.21 % to 10.76 %, then increases to 13.78 %. All demonstrate a pattern of first decrease and then increase. The inflection points for these trends are all observed at MPGF_PVA0.2_. Notably, –OH…O hydrogen bonds continue to increase from 13.70 % to 17.37 %. Therefore, a slight increase in PVA concentration results in the transformation of locally ordered cyclic –OH and free –OH into disordered self-associated –OH and –OH…O. With a further increase in PVA concentration, disordered self-associated –OH transforms back into locally ordered cyclic –OH hydrogen bonds, free –OH, and –OH…O. This indicates that an increase in PVA concentration leads to a microgel with stronger interactions between intermolecular hydrogen bonds. However, at lower PVA concentrations, the enhanced interactions disrupt the ordered structure of hydrogen bonds, while an excess of PVA restores the ordered structure of hydrogen bonds. The variation in hydrogen bond structure explains the changes in the particle size of Ti_3_C_2_T_x_/PVA microgels. In other words, an appropriate PVA concentration can disorder the hydrogen bond structure of Ti_3_C_2_T_x_/PVA microgels. This disruption leads to an increase in volume in a water environment. Consequently, there is an increase in particle size and a broader distribution of particle sizes.

**Fig. 9:**
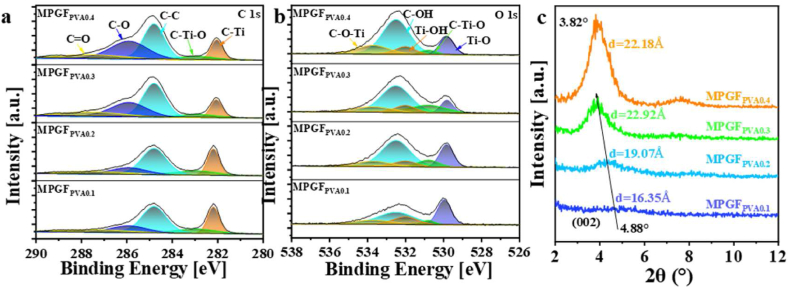
(a) XPS C1s spectra, (b) XPS O1s spectra, and (c) XRD patterns of MPGF_PVA0.1_ to MPGF_PVA0.4_ hydrogels.

In Fig. 9a, the C1s spectrum of Ti_3_C_2_T_x_/PVA hydrogel is shown. With increasing PVA concentration, the peaks at 286.1eV and 284.8eV corresponding to C–O and C–C bonds gradually increase. This indicates a rise in the number of surface C–O and C–C bonds in Ti_3_C_2_T_x_. The binding energy of the C–Ti bond gradually weakens to 282.0eV. This suggests a transfer of valence electrons from the Ti_3_C_2_T_x_ surface to the interior.

Fig. 9b displays the O1s spectrum of the hydrogel. With increasing PVA concentration, the binding energy of Ti–O bonds on the Ti_3_C_2_T_x_ surface decreases from 530.1eV to 529.9eV. The peak of C–OH at 533.4eV gradually strengthens. while the change in the fitting peak of Ti–OH at 532.0eV is not significant. Meanwhile, the change in Ti–OH bond at 532.0eV is not significant. This indicates that the hydrogen bonding interaction between the Ti_3_C_2_T_x_ surface and PVA strengthens with increasing PVA content. It is consistent with the analysis of hydrogen bonds. Thus, at a constant degree of cross-linking, the hydrogen bond structure becomes an important factor influencing the particle size and size distribution of Ti_3_C_2_T_x_/PVA hydrogels.

In Fig. 9c, further XRD analysis was conducted to study the influence of PVA content on the crystal structure of the hydrogel. The intensity of the (002) peak increases with increasing PVA content, indicating an enhancement of the orderliness in Ti_3_C_2_T_x_/PVA hydrogels. Simultaneously, the peak position shifts from 4.88° to a stable 3.82° in MPGF_PVA0.3_, and the interlayer spacing increases from 1.81 nm to 2.31 nm. This demonstrates that increasing PVA concentration intercalation, but excessive addition only further enhances the crystalline structure's orderliness. In summary, increasing PVA concentration can enhance the interaction between Ti_3_C_2_T_x_ and PVA, improving the crystalline structure's orderliness. However, with unchanged cross-linking, the ordered structure of hydrogen bonds becomes a crucial factor affecting the particle size of Ti_3_C_2_T_x_/PVA hydrogels.

In conclusion, by adjusting hydrogen bonds, cross-linking, and the crystal structure in the Ti_3_C_2_T_x_/PVA hydrogel framework, larger particle size and broader distribution can be achieved. This leads to a shorter formation time for the vanadium-based aqueous film.

#### 3.3.3 Microstructure and mechanical properties of MPGF

To examine the influence of the Ti_3_C_2_T_x_/PVA microgel structure and particle size distribution on the microstructure and mechanical properties of self-assembled films, two film preparation methods, namely freeze-drying and hot-drying, were employed.

**Fig. 10:**
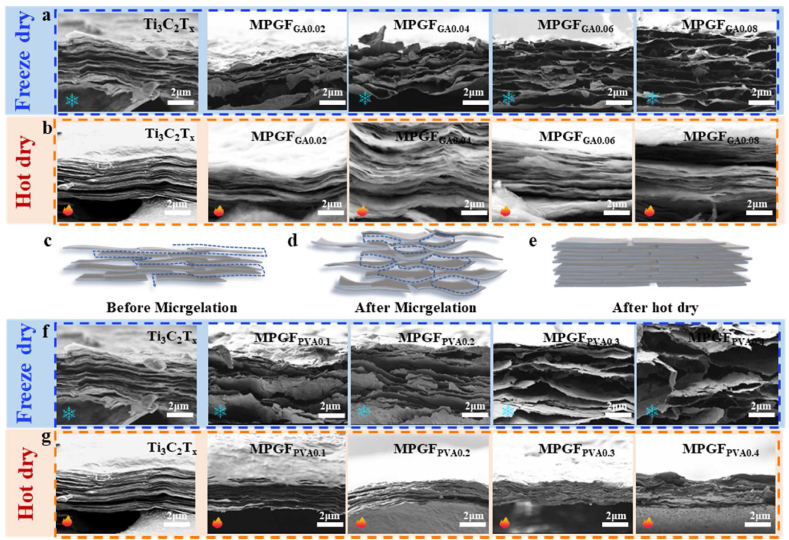
Cross-sectional SEM images of self-assembled films from Ti_3_C_2_T_x_/PVA hydrogels with varying glutaraldehyde dosage: (a) freeze-drying, (b) hot-drying. Schematic representation of the structure (c) before gelation of Ti_3_C_2_T_x_, (d) after gelation, and (e) cross-sectional of the film; Cross-sectional SEM images of Ti_3_C_2_T_x_ and Ti_3_C_2_T_x_/PVA self-assembled films at different PVA concentrations: (f) freeze-drying and (g) hot-drying.

Fig. 10a presents cross-sectional SEM images of freeze-drying Ti_3_C_2_T_x_ and MPGF_GA0.02_ to MPGF_GA0.08_ self-assembled films. Ti_3_C_2_T_x_ displays a dense layered structure, marked by the close stacking of Ti_3_C_2_T_x_ layers "face-to-face." In this compact configuration, water molecules can only permeate through the layer edges. This leads to an extended VAF formation time due to a longer pathway. Conversely, MPGF_GA0.02_ to MPGF_GA0.08_ self-assembled films lack "face-to-face" stacking, providing ample space for mass transfer during water filtration and thereby reducing the VAF formation time (see Fig. 4c). It is crucial to note that an increase in cross-linking results in an expansion of the interlayer spacing in Ti_3_C_2_T_x_/PVA films, but the VAF formation time does not decrease. This discrepancy arises because SEM only reveals changes in interlayer spacing, while factors influencing VAF time encompass the interlayer environment. According to LSPA analysis (see Fig. 4a and b), in the actual water environment of VAF, MPGF_GA0.02_, which has a more disordered hydrogen bond structure, lower crosslinking density, and more disordered crystal structure, is more prone to swelling. This results in higher water permeability and a shorter VAF time.

As depicted in Fig. 10b, the hot-drying Ti_3_C_2_T_x_/PVA film showcases a dense layered structure like the Ti_3_C_2_T_x_ film. This suggests that the Ti_3_C_2_T_x_/PVA microgel framework is essentially a temporary structure. While freeze-drying allows the Ti_3_C_2_T_x_ layers to retain a certain framework structure, hot-drying results in continued tight stacking. Schematic diagrams in Fig. 10c–e elucidate the distinctions in the layered structure arising from the two forming processes. In comparison to Ti_3_C_2_T_x_, the Ti_3_C_2_T_x_/PVA microgel exhibits a shorter water passage and lower resistance to water permeation during the VAF process. After freeze-drying, it retains this passage partially, whereas the Ti_3_C_2_T_x_/PVA film after hot-drying persistently stacks into a dense layered structure.

In Fig. 10f, the cross-sectional SEM images of freeze-drying Ti_3_C_2_T_x_ and MPGF_PVA0.1_ to MPGF_PVA0.4_ are further examined. With an increase in PVA content, the film's interlayer spacing significantly expands. However, when the PVA content exceeds 0.2 wt%, some Ti_3_C_2_T_x_ layers suffer damage, leading to the initiation of a collapse in the layered structure. At 0.4 wt%, extensive damage occurs to the Ti_3_C_2_T_x_ layers. According to FT-IR and XPS analyses (refer to Fig. 8 and 9a and b), this phenomenon occurs because, at high PVA concentrations, Ti_3_C_2_T_x_ primarily establishes connections with PVA through hydrogen bonding. Excessive addition of PVA, attributed to the large volume of PVA molecules, increases the interlayer spacing of Ti_3_C_2_T_x_, and disrupt the Ti_3_C_2_T_x_ layers. Notably, Ti_3_C_2_T_x_/PVA-II, characterized by a more disordered hydrogen bond structure, displays the shortest VAF time, in contrast to Ti_3_C_2_T_x_/PVA-IV, which has the maximum interlayer spacing. This difference arises because the VAF time is affected by both interlayers spacing and the interlayer environment.

In Fig. 10g, the hot-drying MPGF_PVA0.1_ to MPGF_PVA0.4_ still exhibit dense stacking; however, beyond 0.2 wt% PVA content, the layered structure undergoes some degree of damage.

**Fig. 11:**
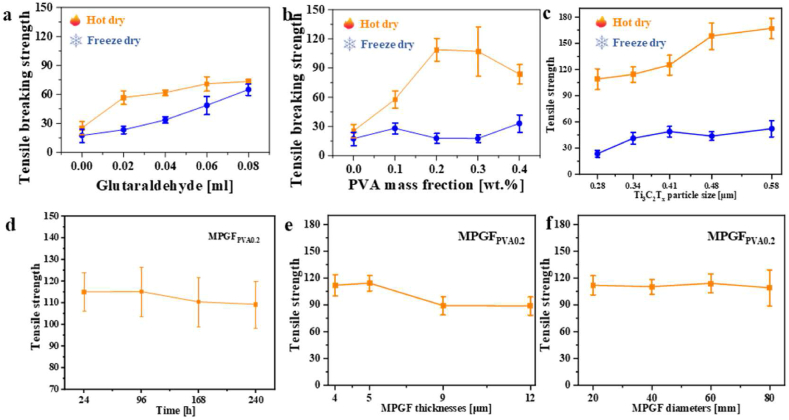
Mechanical properties of the films: (a) Different glutaraldehyde dosage, (b) Different PVA concentrations, (c) Different Ti_3_C_2_T_x_ particle size, (d) Were placed for different durations, (e) Different MPGF thickness, (f)Different MPGF diameters.

Continuing the investigation into the microgel structure and the impact of different drying processes on the mechanical properties of films, Fig. 11a presents the fracture strength variation curves of self-assembled films, including Ti_3_C_2_T_x_, MPGF_GA0.02_ to MPGF_GA0.08_. The blue and orange curves represent films formed by freeze-drying and hot-drying, respectively.

Initially, with an increase in glutaraldehyde content, the fracture strength of the freeze-drying Ti_3_C_2_T_x_/PVA self-assembled film gradually increases to 68 MPa, exhibiting 3.0 times increase compared to the mean of 24 MPa for the Ti_3_C_2_T_x_ film. This enhancement is attributed to the gradual ordering of hydrogen bonds, cross-linking, and crystal structures within the film as the cross-linking density increases, as evidenced by studies involving FT-IR, XPS, and XRD. In contrast, the hot-drying Ti_3_C_2_T_x_/PVA film shows a significant strength increase, reaching 56 MPa first and then slowly rising to 72 MPa. The difference between the hot-drying and freeze-drying films is more significant at lower cross-linking densities and diminishes as cross-linking density increases. This is because the hot-drying film has a dense layered structure similar to the Ti_3_C_2_T_x_ film, and at lower cross-linking densities, the large PVA molecules between the layers play a major reinforcing role. When cross-linking density is lower, the film's intramolecular self-associated –OH hydrogen bond content is at its highest, reinforcing the intramolecular interaction of PVA. With increased cross-linking density, the film's hydrogen bonds, cross-linking, and crystal structure all become more ordered, the strength of the freeze-drying film has also gradually been enhanced.

Continuing the study on the influence of PVA quantity on film mechanical properties, the fracture strength of Ti_3_C_2_T_x_, MPGF_PVA0.1_ to MPGF_PVA0.4_ is depicted in Fig. 11b. With an increase in PVA quantity, there is a substantial difference in the mechanical properties of freeze-drying and hot-drying films compared to Ti_3_C_2_T_x_. The freeze-drying film's strength changes are less noticeable, and regardless of PVA quantity, the fracture strength remains below 30 MPa. This is because the freeze-drying film's layered structure is not dense enough, and under constant cross-linking, excessive PVA intercalation tends to damage some Ti_3_C_2_T_x_ layers within the film, as observed in SEM studies.

In contrast, with an increase in PVA quantity, the fracture strength of the hot-drying film first dramatically increases to 112 MPa and then slowly decreases to 82 MPa, with the maximum occurring at the MPGF_PVA0.2_. The fracture strength of the Ti_3_C_2_T_x_/PVA self-assembled film increases up to 4.7 times compared to the Ti_3_C_2_T_x_ film. In comparison to the freeze-drying film, the mechanical performance of the hot-dryingfilm, with its dense layered structure, primarily depends on PVA reinforcement. The MPGF_PVA0.2_, with the highest content of intramolecular self-associated –OH hydrogen bonds, reinforces the effect of PVA. However, based on FT-IR, XPS, and XRD studies, although the intermolecular interactions, hydrogen bonds, and crystalline structure orderliness in the Ti_3_C_2_T_x_/PVA self-assembled film increase with an increase in PVA quantity, SEM analysis of the film cross-section reveals that the slow decrease in fracture strength is due to the disruption of Ti_3_C_2_T_x_ layers. Therefore, Therefore, the integrity of Ti_3_C_2_T_x_ layers is crucial to ensuring the mechanical performance of the membrane.

The presence of a dense layered structure renders the influence of PVA on film performance significant. As the PVA content increased to 0.2 wt%, the cyclic –OH hydrogen bonds and free –OH hydrogen bonds within the film transitioned to intramolecular self-bonding –OH hydrogen bonds, resulting in their content increasing to 62.56 % and enhancing the internal cohesion of PVA within the film, leading to a fracture strength of 112 MPa. However, with further increases in PVA content, the content of self-bonding –OH hydrogen bonds began to decrease, reaching 55.17 % at a content of 0.4 wt%, and the content of free –OH exceeded that before the transition. SEM characterization results indicated that at this point, excessive PVA intercalation led to localized damage to the layered structure, ultimately resulting in a decrease in film fracture strength to 82 MPa. Therefore, to enhance the mechanical performance of the film, a balance must be struck between moderate PVA intercalation and a rational hydrogen bond structure.

We obtained Ti_3_C_2_T_x_ sheets of different sizes by varying the ultrasonication time during the preparation of the Ti_3_C_2_T_x_ colloidal solution, and prepared corresponding MPGF, testing their mechanical properties. The results indicate that increasing the size of the Ti_3_C_2_T_x_ sheets further enhances the mechanical performance of the films. Therefore, in the revised manuscript, we have added the following discussion: "Reducing the ultrasonication time during the preparation of the Ti_3_C_2_T_x_ colloidal solution resulted in obtaining larger-sized Ti_3_C_2_T_x_ sheets. As shown in Figs. S2a, b, c, d, e, with ultrasonication times of 60, 50, 40, 30, and 20 min, the median particle size of Ti_3_C_2_T_x_ sheets was measured as 0.28 μm, 0.34 μm, 0.41 μm, 0.48 μm, and 0.58 μm, respectively. Subsequently, using the same glutaraldehyde and PVA ratio as MPGF_PVA0.2_, MPGF with different Ti_3_C_2_T_x_ sizes were prepared. The mechanical properties of these films, as shown in Fig. 11c, revealed that with an increase in Ti_3_C_2_T_x_ sheets size, the fracture strength of the dried films increased from 112 MPa to 167 MPa, representing a 0.5-fold enhancement, while the fracture strength of the freeze-dried films increased from 23 MPa to 52 MPa. This enhancement could be attributed to the reduced voids or defects generated during the stacking of larger Ti_3_C_2_T_x_ sheets during the self-assembly process. Further reduction in ultrasonication time, as depicted in Fig. S3, resulted in a bimodal distribution of Ti_3_C_2_T_x_ sheets, indicating incomplete exfoliation of the Ti_3_C_2_T_x_ sheets.

The study indicates that MXene films are prone to oxidation, resulting in the loss of their mechanical properties. Fig. S4 displays the TG curve of MPGF_PVA0.2_ in an oxygen environment. The temperature ranges from room temperature to 600 °C, and the weight change occurs in three steps. The first step involves the release of adsorbed free water, the second step is attributed to the release of surface-bound water and functional groups, and the third step is due to the decomposition of PVA. However, no weight gain was observed, whereas weight gain would be expected during MXene oxidation. This suggests that within 600 °C, the film did not undergo oxidation. Fig. 11d presents the mechanical performance data of MPGF_PVA0.2_ after exposure to air for 1, 4, 7, and 10 days. Before the 7th day, the tensile fracture strength of the film remained stable around 116 MPa, slightly decreasing after the 7th day, and further decreasing to only 109 MPa after the 10th day. This indicates that MPGF_PVA0.2_ exhibits a certain degree of stability in air.

The thickness and lateral size of the film also significantly affect its performance. Fig. S5 shows the cross-sectional SEM images of films with different thicknesses, and Fig. 11e and f shows the fracture strength data of MPGF_PVA0.2_ with different thicknesses and diameters. It can be observed that when the film thickness exceeds 8.5 μm, the fracture strength begins to decrease, and when the thickness reaches 12.3 μm, the mechanical performance decreases to 89 MPa. SEM images show that thicker films exhibit increased internal folds in the layered structure, leading to stress concentration and a decrease in mechanical performance. The impact of film diameter on mechanical performance is relatively small, with a slight decrease in fracture strength to 109 MPa when the film diameter reaches 80 mm. However, it is worth noting that the test results for the fracture strength of films with larger diameters become unstable, with an increase in standard deviation, because the excessively large diameter causes uneven deposition of the microgel in the horizontal direction during the VAF process. Therefore, excessive thickness leads to microscale wrinkles and defects in the film, reducing its mechanical performance, while excessive increase in the lateral size of the film reduces its uniformity, thereby reducing the stability of the film's mechanical performance.

In conclusion, there is a significant difference in the mechanical performance between freeze-drying and hot-dryingfilms, and increasing cross-linking density can reduce this difference, enhancing the mechanical performance of freeze-drying films. However, excessive addition of PVA at constant cross-linking density is not conducive to improving mechanical performance. The mechanical properties of hot-dryingfilms are mainly influenced by PVA, and PVA intercalation and the generation of intramolecular self-associated –OH hydrogen bonds contribute significantly to a substantial improvement in film mechanical performance. However, all of this is contingent upon the integrity of Ti_3_C_2_T_x_ layers.

The formation of a microgel framework guided by glutaraldehyde crosslinking has been shown to enhance the preparation efficiency and mechanical performance of MXene/PVA composite films, which is important for practical production and application. We also investigated the effects of glutaraldehyde dosage, PVA dosage, MXene sheet size, and film size on the mechanical properties of the films, thereby elucidating the relationship between film structure and performance under this self-assembly mechanism. This provides detailed data support and theoretical foundation for subsequent practical application research, advancing the laboratory research of MXene-based films towards industrialization and real-world applications.

## 4 Conclusions

This study delves into the rapid preparation, structure, and properties of Ti_3_C_2_T_x_/PVA self-assembled films, leading to the following key findings.

(1) Microgel Framework Formation: FT-IR, XPS, and XRD analyses disclose that glutaraldehyde crosslinking initiates the development of a microgel framework between Ti_3_C_2_T_x_ and PVA. In this framework, PVA layers are intricately inserted between Ti_3_C_2_T_x_ layers, differentiating it from a conventional Ti_3_C_2_T_x_/PVA intercalation structure.
(2) Prevention of Layer Stacking: SEM and TEM analyses illustrate that Ti_3_C_2_T_x_/PVA microgels effectively inhibit the "face-to-face" stacking of Ti_3_C_2_T_x_ layers during Vacuum-Assisted Filtration (VAF), establishing pathways for water permeation.
(3) Particle Size Impact: LSPA analysis and VAF molding time statistics demonstrate a significant increase in the particle size of Ti_3_C_2_T_x_/PVA microgels compared to Ti_3_C_2_T_x_. This increase leads to a substantial reduction in the fastest VAF molding time, decreasing from 90400s to an impressive 69 s.
(4) Structural Transformation: Structural analysis indicates that heightened crosslinking promotes the transformation of intramolecular self-complementary –OH hydrogen bonds into intermolecular OH⋯O hydrogen bonds and locally ordered cyclic –OH hydrogen bonds. This transformation enhances the order of hydrogen bond structures and crystal structures, albeit with a slight increase in VAF molding time.
(5) Effect of PVA Content: Structural analysis suggests that an increasing PVA content continuously enhances OH⋯O hydrogen bond interactions and interlayer distance of microgels. However, at a concentration of 0.2 wt%, the cyclic –OH hydrogen bond content is the lowest, resulting in the most disordered hydrogen bond structure. Consequently, the microgel becomes highly prone to swelling, leading to the shortest VAF molding time.
(6) Microstructure Insights: Microstructure analysis of Ti_3_C_2_T_x_/PVA self-assembled films reveals that Ti_3_C_2_T_x_/PVA microgels form a temporary 3D framework. During freeze-drying, the film retains some microgel space, while hot-drying results in a dense layered structure. Increasing crosslinking and PVA intercalation contribute to enhancing the microstructure's order. However, excessive PVA content can disrupt Ti_3_C_2_T_x_ layers.
(7) Mechanical Property Evaluation: Mechanical property analysis indicates that in freeze-drying films, characterized by a non-dense micro-layered structure, mechanical properties are primarily influenced by hydrogen bonds, crosslinking, and the order of crystal structures. A well-ordered structure leads to impressive mechanical properties, reaching a maximum of 68 MPa, three times that of Ti_3_C_2_T_x_ films. Conversely, hot-drying films, with a dense micro-layered structure, are mainly influenced by intercalated PVA. High content of intramolecular self-complementary –OH hydrogen bonds contribute to good mechanical properties, reaching a maximum of 112 MPa, 4.7 times that of Ti_3_C_2_T_x_ films. However, it is crucial to ensure the integrity of Ti_3_C_2_T_x_ layers for these improvements.

## CRediT authorship contribution statement

**Ziwen Gan:** Writing – review & editing, Methodology, Investigation, Formal analysis, Data curation, Conceptualization. **Ranran Qi:** Writing – review & editing, Validation, Methodology, Formal analysis. **Bowen Chen:** Validation, Methodology, Formal analysis. **Gaofei Yuan:** Validation. **Mingyi Liao:** Writing – review & editing, Supervision, Funding acquisition.

## Declaration of competing interest

The authors declare that they have no known competing financial interests or personal relationships that could have appeared to influence the work reported in this paper.

## Appendix A Supplementary data

Multimedia component 1 Multimedia component
1
