# Supplementary material for: Ultra-fast fabrication of MXene/PVA composite films through glutaraldehyde induced microgel framework
Source: Heliyon. 2024 May 6;10(9):e30714. doi: 10.1016/j.heliyon.2024.e30714 (PMC11110175; doi:10.1016/j.heliyon.2024.e30714)
Supplement: Multimedia component 1 [file mmc1.docx]

**1. TG analysis of MPGF**

Figure S1 shows the TG curves of MPGF films in a nitrogen atmosphere. The temperature ranges from room temperature to 600°C. The weight change is divided into three steps. The first step is due to the release of surface adsorbed free water, the second step is due to the release of surface-bonded water and functional groups, and the third step is due to the decomposition of PVA. It can be seen that the addition of glutaraldehyde, the decomposition temperature of free water for MPGFG0.02, MPGFG0.04, and MPGFG0.06 remains almost unchanged at 49.68±0.5°C, and the content also remains unchanged at 8.51±0.6%, indicating that the addition of glutaraldehyde in this dosage range does not affect the binding of the film to free water, with minimal changes in hydrophilicity. Furthermore, the decomposition temperature and content of bonded water and active functional groups for MPGF_GA0.02_, MPGF_GA0.04_, and MPGF_GA0.06_ also show minimal changes, at 196.59±4°C and 6.52±0.07%, respectively, indicating that at this dosage, the consumption of active functional groups by glutaraldehyde remains at a low level, with minimal overall impact on the film's activity. Additionally, there are minimal changes in the decomposition temperature and content of PVA, with the lowest decomposition temperature at 417.87±2.93°C, the highest decomposition temperature at 523.73±7.74°C, and a content change from 3.73±0.17%, indicating that the consumption of PVA by glutaraldehyde at this dosage level is also minimal, with minimal impact on the overall composition and structure of the film. However, it is worth noting that there are significant changes in the decomposition temperature and content of bonded water and active functional groups for MPGF_GA0.08_, increasing from 193.05°C to 212.58°C and decreasing from 6.54% to 2.21%, respectively, indicating that excessive addition of glutaraldehyde leads to significant consumption of active functional groups in the film, but contributes to the improvement of thermal stability of the remaining active functional groups. Additionally, the lowest decomposition temperature of PVA decreases from 419.07°C to 376.47°C, the highest decomposition temperature increases from 531.05°C to 545.76°C, and the content decreases from 3.59% to 3.17%, indicating that excessive addition of glutaraldehyde increases the consumption of PVA, polarizing the internal components and structure of the film, which may affect the film's performance.


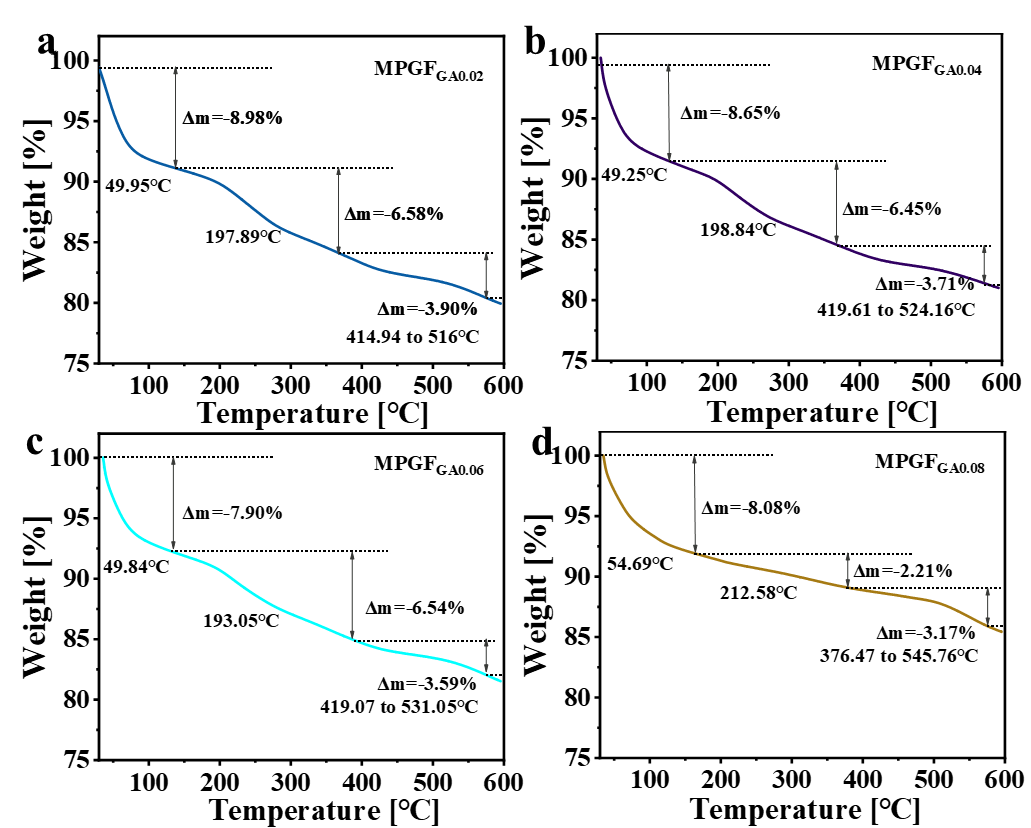


**Figure S1. The TG curves of MPGF_GA0.02_, MPGF_GA0.04_, MPGF_GA0.06_, and MPGF_GA0.08_ in a nitrogen atmosphere.**


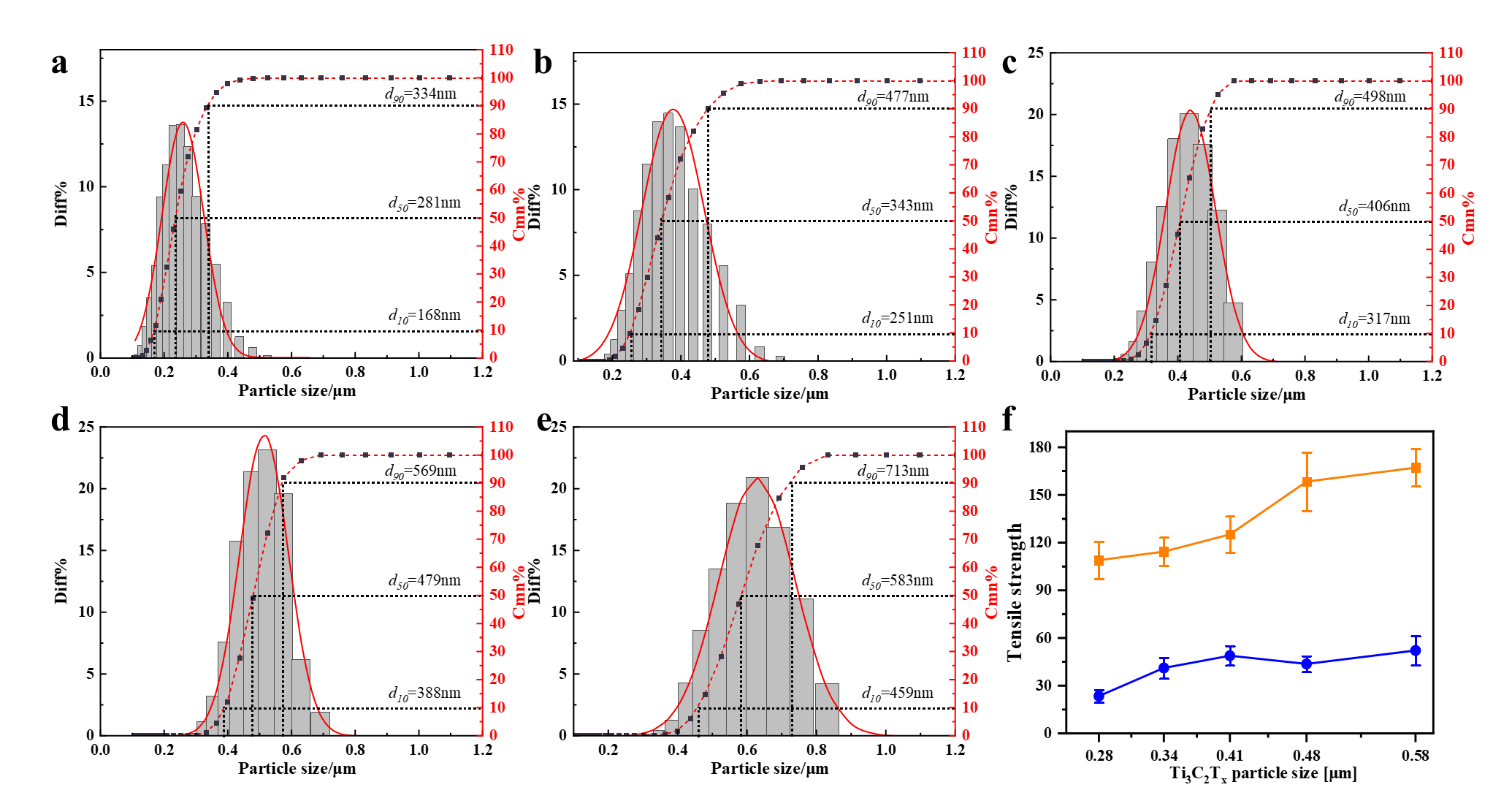


**Figure S2a-e. The particle size distribution curves of Ti_3_C_2_T_x_ sheets at ultrasonication times of 60 min, 50 min, 40 min, 30 min, and 20 min, respectively; 2f shows the tensile fracture strength curves of MPGF prepared from Ti_3_C_2_T_x_ sheets of different sizes.**


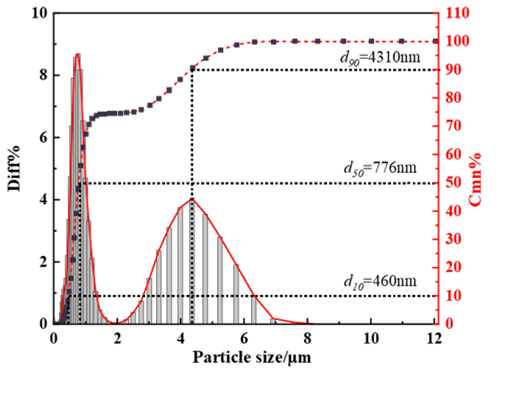


**Figure S3. The particle size distribution curves of Ti_3_C_2_T_x_ sheets at ultrasonication times of 10 min.**


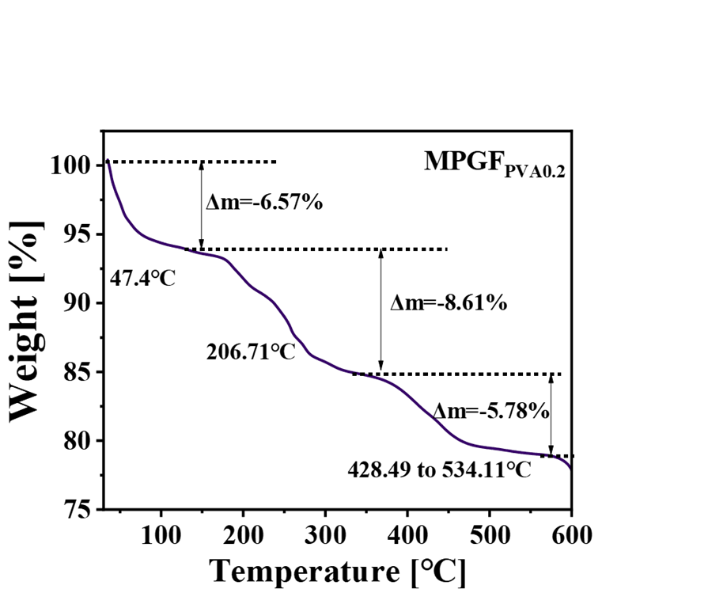


**Figure S4. TG curve of MPGF_PVA0.2_ in an oxygen environment.**


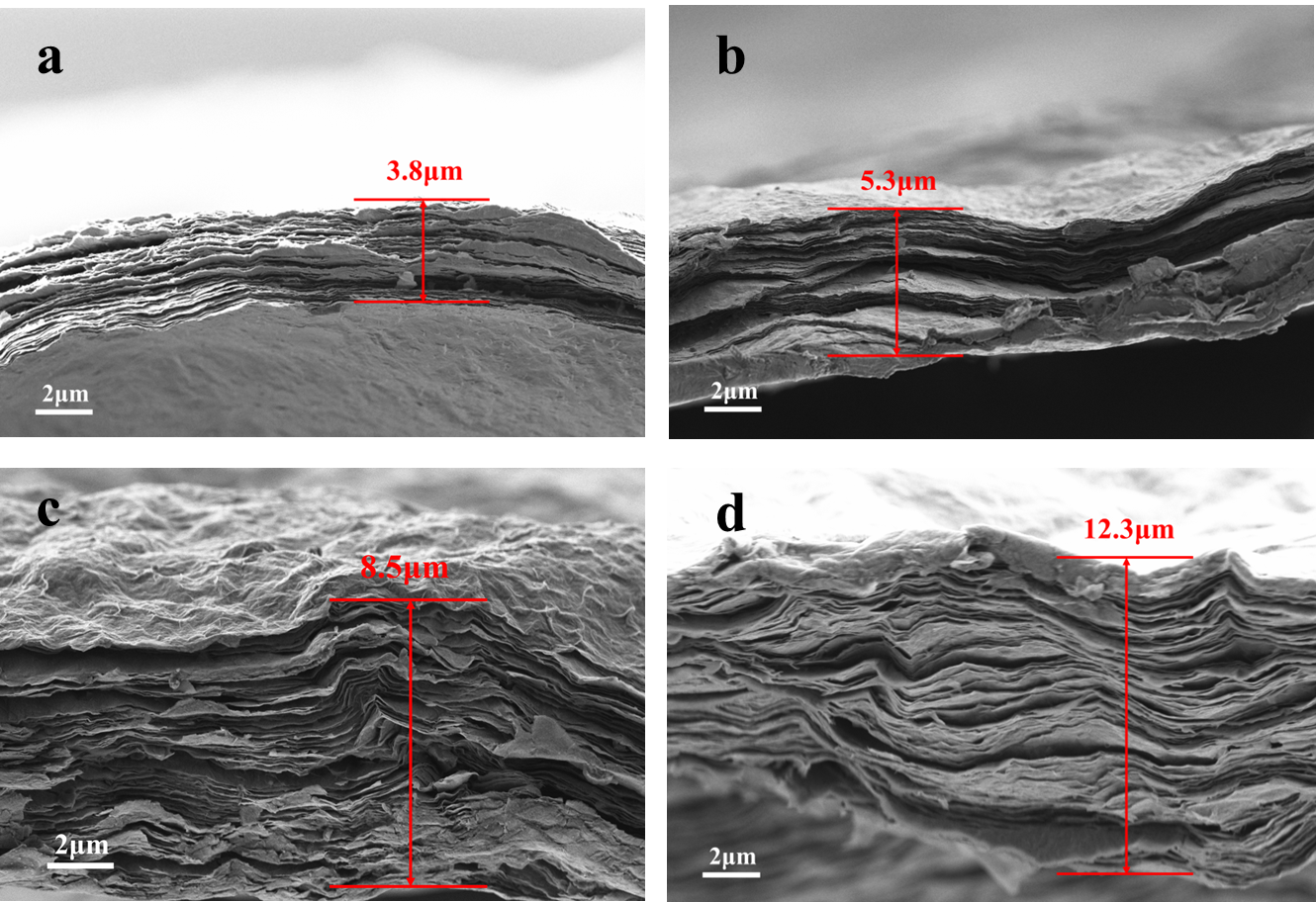


**Figure S5. SEM images of cross-sections of MPGFs with different thicknesses.**

**Table S1. Detailed VAF time data for several types of MXene films**.

|  | **VAF time [s]** | **Mean [s]** | **Standard deviation** |
| --- | --- | --- | --- |
| Ti_3_C_2_T_x_ film | 58483 | 90400 | 1523 |
|  | 61874 |  |  |
|  | 62038 |  |  |
|  | 59921 |  |  |
|  | 59684 |  |  |
| Ti_3_C_2_T_x_/GA film | 23991 | 24493 | 398 |
|  | 24239 |  |  |
|  | 24816 |  |  |
|  | 24953 |  |  |
|  | 24466 |  |  |
| Ti_3_C_2_T_x_/PVA film | 17559 | 17807 | 535 |
|  | 18223 |  |  |
|  | 18265 |  |  |
|  | 17998 |  |  |
|  | 16992 |  |  |
